# Graded ankle instability induces early osteoarthritic changes in a rat model

**DOI:** 10.1016/j.ocarto.2026.100784

**Published:** 2026-05-02

**Authors:** Kenji Murata, Sora Kawabata, Chiharu Takasu, Nihei Kota, Naoki Shimada, Naohiko Kanemura

**Affiliations:** a Department of Physical Therapy, School of Health and Social Services, Saitama Prefectural University, Saitama, Japan; b Department of Health and Social Services, Health and Social Services, Graduate School of Saitama Prefectural University, Saitama, Japan

**Keywords:** Osteoarthritis, Joint instability, Ankle sprain, Ligament injury

## Abstract

**Objective:**

To use a rat model of graded ankle instability and mechanical laxity observed in ankle sprains to examine early cartilage degeneration and explore molecular alterations.

**Design:**

Graded ankle instability was induced by transection of the anterior talofibular and calcaneofibular ligaments, resulting in the following experimental groups: sham-operated control group; transected calcaneofibular ligament group; and combined transected anterior talofibular and calcaneofibular ligament group. Radiographic and histological analyses were performed at 4, 6, and 8 weeks postoperatively, and functional performance was assessed using the ladder walking test. Moreover, an exploratory RNA sequencing analysis of ankle cartilage was conducted to identify differentially expressed genes (DEGs) in early osteoarthritic and normal cartilage. Gene Ontology and network analyses were conducted using a Gene Set Enrichment Analysis.

**Results:**

The anterior drawer distance and talar tilt angle increased proportionally with ligament transection, thus confirming graded mechanical instability. The histological evaluation showed severity-dependent cartilage degeneration with increased Osteoarthritis Research Society International scores associated with the degree of instability. Exploratory RNA sequencing identified 245 significant DEGs (132 upregulated and 113 downregulated) with macrophage-related gene enrichment. Among these, CCL2 and TRPV4 with osteoarthritis progression were highlighted as existing genes that potentially link mechanical stress to inflammatory responses in cartilage.

**Conclusion:**

This graded ankle instability model allowed an experimental evaluation of relationships between joint instability, early cartilage degeneration, and associated molecular changes.

## 1 Introduction

Ankle sprains are among the most common musculoskeletal injuries sustained during sports activities and daily life [1]. The severity of ligament injury is clinically graded and ranges from stretching to complete rupture. The degree of ligament injury is closely associated with the extent of ankle joint instability, and a subset of patients develop chronic ankle instability (CAI) [2]. CAI is characterized by recurrent sprains, episodes of “giving way,” and persistent joint laxity. Such mechanical instability alters the joint loading environment and induces abnormal stress distribution, which may ultimately accelerate cartilage degeneration.

Joint instability is a key pathological factor involved in the onset and progression of post-traumatic osteoarthritis (PTOA) [3,4]. In the knee joint, mechanical instability following ligament injury promotes PTOA progression. Studies by Tochigi [5], Murata [6,7], and others have reported a quantitative correlation between the degree of joint instability and severity of cartilage degeneration, suggesting that altered joint mechanics after ligament injury greatly contribute to degenerative changes. PTOA models of the knee joint, including the anterior cruciate ligament transection model [8,9] and medial meniscus destabilization model [10], have been more readily established and widely used to study disease mechanisms. In contrast, ankle osteoarthritis studies have largely used models involving chemical induction, such as monoiodoacetate injection [[11], [12], [13]], which mainly reproduce acute metabolic cartilage damage rather than gradual mechanically driven degeneration following ligament injury.

Recently, we reported [14] ankle instability induced by transection of the calcaneofibular ligament (CFL) alone and demonstrated pain-related and ligament function alterations following ligament injury. Additionally, several surgical ankle injury models involving rodents, such as mouse models including transection of the anterior talofibular ligament (ATFL) and CFL, have been reported [15,16]. Although these studies successfully reproduced ligament injury-related changes and reported cartilage degeneration and subchondral bone alterations over longer follow-up periods, quantitative evaluations of early osteoarthritic changes and analyses that correlated the degree of mechanical instability with cartilage degeneration were not performed. The early phase of OA progression between graded ankle instability and cartilage degeneration has not been characterized by previous studies.

In this study, we established a rat model of graded ankle instability by sequentially transecting the ATFL and CFL to mimic mild and severe ligament injury. We examined how the severity of instability affects histological changes (cartilage degeneration) and radiographic findings (joint alignment and instability). This study aimed to explore how ankle instability contributes to cartilage degeneration and provide insights relevant to the understanding of early PTOA.

## 2 Materials and methods

### 2.1 Animals

Forty-five male Wistar rats (4 weeks old) were obtained from Sankyo Lab Service Co., Inc. (Tokyo, Japan) and acclimated to the laboratory environment for 7 days before the start of the experiment. Rats were housed in pairs in polypropylene cages under controlled environmental conditions (temperature, 24°; relative humidity, 55%; 12-h light/dark cycle). Automatic watering systems were provided. Animals had free access to standard laboratory chow and water ad libitum. All study procedures were approved by the animal research committee of Saitama Prefectural University (approval no. 2021-1).

### 2.2 Surgical procedure

All animals were randomly divided into the following three groups comprising 15 rats per group: mild ankle instability (MAI) group; severe ankle instability (SAI) group; and sham group. Group allocation was performed by an independent researcher (S.K.) who was blinded to the group identity at the time of allocation to minimize allocation bias. Under inhalation anesthesia with isoflurane (2%–3% in oxygen), anesthetic comprising medetomidine (0.375 mg/kg), midazolam (2.0 mg/kg), and butorphanol (2.5 mg/kg) was administered. Before surgery, the right hind limb was shaved and disinfected with povidone-iodine solution. A longitudinal incision was created over the lateral malleolus to expose the ATFL and CFL. In the MAI group, only the CFL was transected using micro-scissors. In the SAI group, both the ATFL and CFL were transected. In the sham group, the ligaments were exposed but not cut. After each procedure, the wound was rinsed with sterile saline and closed using 4–0 nylon sutures. The surgical procedure only included extra-articular ligament injury with a minimal incision; therefore, no additional postoperative analgesics were administered. Postoperative immobilization and external fixation were not applied. Animals were allowed unrestricted cage activity immediately after recovery from anesthesia. Their body weight, gait, and wound condition were monitored daily. Adverse events, including infection, wound dehiscence, mortality, and impaired ambulation, were not observed in any group.

### 2.3 Evaluation of ankle stability

Ankle joint instability was evaluated using a custom-made device designed for rats and both the anterior drawer test (ADT) and talar tilt test (TTT) at 4, 6, and 8 weeks postoperatively by a second author (S.K.) who was blinded. During the ADT, radiographs were obtained using an M − 60 radiographic system (Softex Corporation, Kanagawa, Japan) under the conditions of 30 kVp, 1.5 mA, and 1 s while a constant anterior traction load was applied. The anterior drawer rate was calculated by analyzing relative displacement between the distal tibial and talar articular surfaces using ImageJ software (National Institutes of Health, Bethesda, MD, USA). During the TTT, the ankle was fixed in the inversion position, and radiographs were obtained while a constant lateral load was applied. The talar tilt angle was measured as the angle between the inferior articular surface of the tibia and superior trochlear surface of the talus.

### 2.4 Behavioral tests

Ankle sensorimotor function was evaluated using the ladder walking test. Animals crossed a ladder with a length of 100 cm and walking path width of 4 cm while 2-cm ladder intervals were filmed from the left side in the direction of travel. These tests were performed in accordance with previously described methods [14].

### 2.5 Histological analysis

At 4, 6, and 8 weeks postoperatively, the right ankle joints were harvested (n = 5 per group), fixed in 4% paraformaldehyde, and decalcified in 10% ethylenediaminetetraacetic acid solution. Then, the specimens were embedded in paraffin. Serial sagittal sections (thickness, 5 μm) including both the tibial and talar articular surfaces were prepared and stained with Safranine O/Fast green. Cartilage degeneration was evaluated according to the Osteoarthritis Research Society International (OARSI) scoring system [17,18]. For each knee sample, the scores of three sections independently assessed by two blinded observers were averaged. Interobserver agreement was evaluated using Cohen's kappa coefficient. When minor discrepancies occurred, the mean of the scores determined by the two observers was used for the statistical analysis.

### 2.6 RNA sequencing analysis of articular cartilage

We categorized six 4-week-old male Wistar rats into the SAI group (n = 3) with ankle instability and increased mechanical stress on the articular cartilage in the tibiotalar joint and the sham group (n = 3). Then, we harvested the tibiofemoral joint cartilage and performed RNA sequencing 8 weeks postoperatively to confirm cartilage degeneration. The quality and integrity of the extracted RNA were assessed using a Bioanalyzer (Agilent 2100; Agilent Technologies, Tokyo, Japan). Samples that met the RNA integrity number threshold for high-quality RNA were selected for library preparation using the NEBNext Ultra II RNA Library Prep Kit (cat. no. 7770; Illumina, Inc., San Diego, CA, USA). The libraries were sequenced on an Illumina NovaSeq X Plus (Illumina, Inc., USA) platform to generate paired-end reads.

**Fig. 1:**
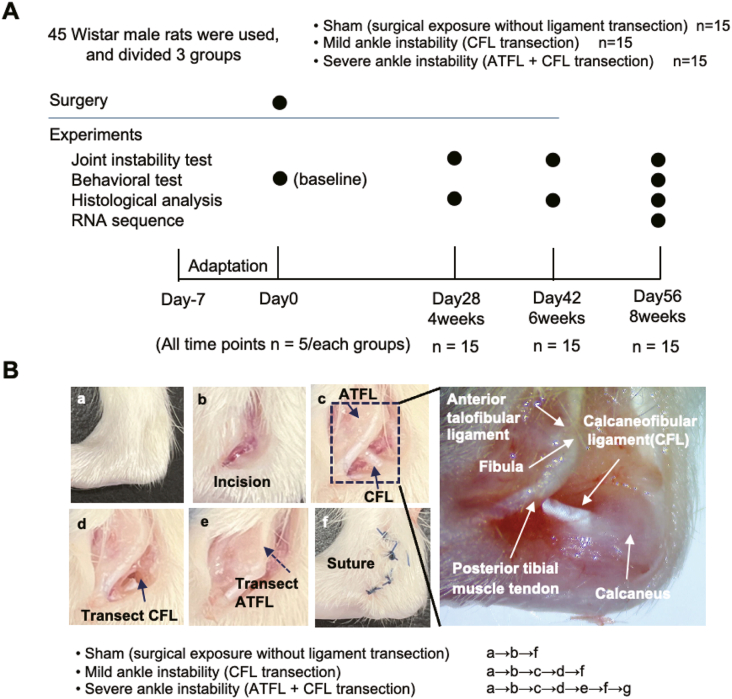
Study design and surgical steps of the rat model of ankle instability. (A) Experimental timeline and study design. Male Wistar rats were assigned to one of three groups: sham surgery, mild ankle instability (calcaneofibular ligament [CFL] transection), or severe ankle instability (combined anterior talofibular ligament [ATFL] and CFL transection). Joint instability testing, behavioral assessment, histological analysis, and RNA sequencing were performed at the indicated postoperative time points (4, 6, and 8 weeks). (B) Representative images illustrating the surgical procedure.

Transcripts per million were calculated based on the gene expression levels, and differentially expressed genes of the two groups were extracted using the DESeq2 package (R Foundation, Vienna, Austria) (p < 0.05) (Fig. 1). Using the extracted differentially expressed genes, we constructed a protein–protein interaction network using STRING (https://string-db.org/) and performed a network analysis using Cytoscape. Additionally, a functional enrichment analysis of Gene Ontology biological processes was conducted using a Gene Set Enrichment Analysis based on the transcripts per million for each sample.

### 2.7 Statistical analysis

Statistical analyses were performed using parametric or nonparametric tests, depending on the data distribution. Data normality was assessed using the Shapiro–Wilk test. For parametric data (ankle instability and behavioral test results), a one-way analysis of variance was conducted, followed by Tukey's post hoc test. For nonparametric data (OARSI score), the Kruskal–Wallis test was conducted, followed by Bonferroni's multiple comparison test. The relationships between ankle instability parameters and OARSI scores were evaluated using Pearson's correlation analysis. Parametric data are presented as bar graphs with individual data points, whereas nonparametric data are shown as scatter plots with individual values and median lines; p < 0.05 was considered statistically significant.

## 3 Results

### 3.1 Ankle joint instability

**Fig. 2:**
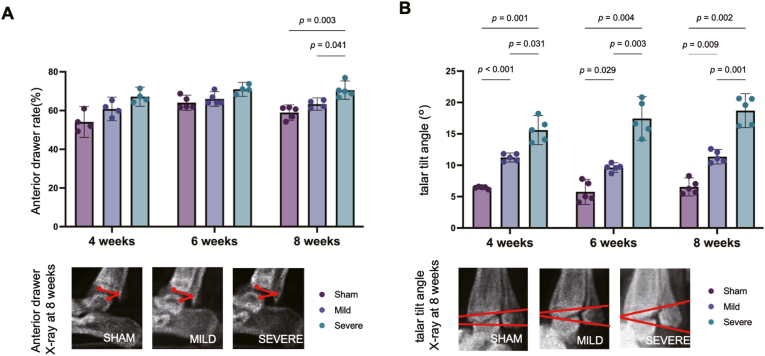
Results of ankle joint instability after injury using the radiographic evaluation across three experimental groups (sham, mild ankle instability [MAI], and severe ankle instability [SAI]) are shown. The MAI and SAI groups increased narrowing of the anterior drawer and talar tilt angle compared with those of the sham group. Statistical analysis was performed using a one-way ANOVA followed by Tukey's post hoc test. Bars indicate the mean ± 95% CI.

Ankle joint stability was quantitatively evaluated using the ADT and TTT (Fig. 2). At 8 weeks postoperatively, the ADT revealed the following stepwise increase in joint instability: sham group, 58.94 (mean; 95% confidence interval, 54.90–62.98); MAI group, 63.30 (mean; 95% CI, 61.15–65.45); and SAI group, 70.54 (mean; 95% CI, 65.80–75.28). The SAI group exhibited significantly greater instability than that in the sham group (p = 0.003) and MAI group (p = 0.041). Similarly, at 8 weeks postoperatively, the TTT demonstrated the following stepwise increase in joint instability: sham group, 6.55° (mean; 95% CI, 5.11–7.98); MAI group, 11.37° (mean; 95% CI, 10.19–12.54); and SAI group, 18.70° (mean; 85% CI, 15.98–21.41). The SAI group exhibited significantly greater instability than that in the sham group (p = 0.001) and MAI group (p = 0.009). Notably, significantly higher TTT values in the SAI group compared with those in the MAI group and sham group were evident at 4 weeks and 6 weeks postoperatively (4 weeks: p < 0.001 and p = 0.031; 6 weeks: p = 0.029 and p = 0.004) (Fig. 2).

### 3.2 Functional evaluation

**Fig. 3:**
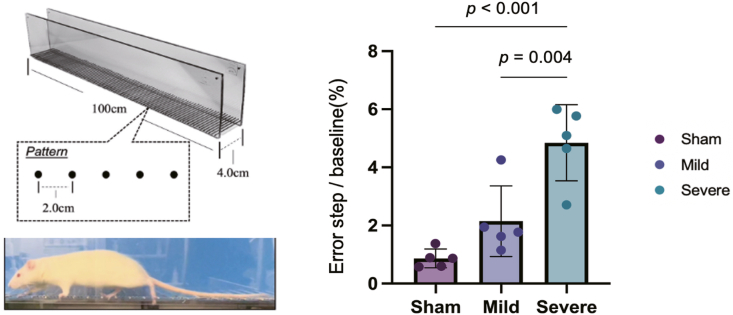
Results of the evaluation of sensorimotor function using the ladder walking test. Each dot represents an individual animal. Statistical analysis was performed using a one-way analysis of variance followed by Tukey's post hoc test. Bars indicate the mean ± 95% CI.

Ankle joint function was evaluated using the ladder walking test (Fig. 3). The error step rate showed the following stepwise increase: sham group, 0.86 (mean; 95% CI, 0.46–1.27); MAI group, 2.15 (mean; 95% CI, 0.64–3.66); and SAI group, 4.85 (mean; 95% CI, 3.22–6.47). The SAI group exhibited a significantly higher error rate than that in the sham group (p < 0.001) and MAI group (p = 0.004).

### 3.3 Histological cartilage degeneration

**Fig. 4:**
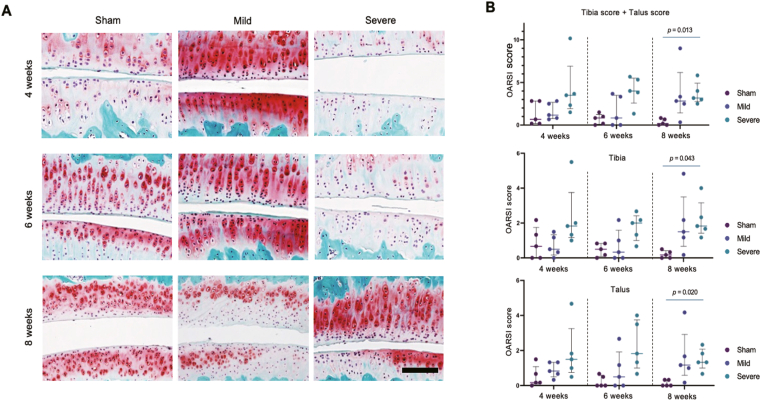
Histological assessment of cartilage degeneration using safranine O staining of the rat ankle joint. (A) Representative sections of the tibiotalar cartilage from the sham, MAI, and SAI groups stained with toluidine blue at 4, 6, and 8 weeks postoperatively. (B) Quantitative histological grading of the tibial surface, talar surface, and combined total score based on the OARSI scoring system. Statistical analysis was performed using Kruskal–Wallis test was conducted, followed by Bonferroni's multiple comparison test. Bars indicate the median and interquartile range.

Interobserver agreement was evaluated using Cohen's kappa coefficient, which showed good agreement (κ = 0.71). Histological scoring revealed significantly higher total OARSI scores (tibia and talus) in the SAI group at 8 weeks postoperatively (median, 3.17; interquartile range, 2.75–4.92) compared with those in the sham group (median, 0.17; interquartile range, 0.00–0.75; p = 0.013), indicating advanced cartilage degeneration (Fig. 4).

### 3.4 Correlation between joint instability and cartilage degeneration

**Fig. 5:**
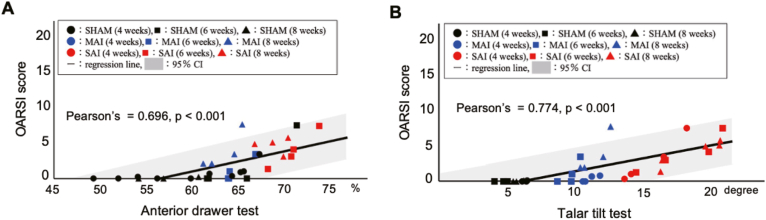
Correlation between joint instability parameters and cartilage degeneration. (A) A significant positive correlation was observed between the anterior drawer rate and OARSI score (Pearson's r = 0.696; 95% CI, 0.463–0.839; p < 0.001). (B) Similarly, the talar tilt angle was positively correlated with the OARSI score (Pearson's r = 0.774; 95% CI, 0.590–0.881; p < 0.001). Each symbol represents an individual sample from the sham, MAI, and SAI groups at 4, 6, and 8 weeks. Shaded areas indicate the 95% CI of the regression line.

To examine the relationship between joint instability and cartilage degeneration, correlation analyses of instability parameters (ADT and TTT) and OARSI scores were performed (Fig. 5). The anterior drawer rate was significantly positively correlated with the total OARSI score (r = 0.696; 95% CI, 0.463–0.839; p < 0.001). Similarly, the talar tilt angle was strongly positively correlated with the OARSI score (r = 0.774; 95% CI, 0.590–0.881; p < 0.001).

### 3.5 RNA sequencing analysis

**Fig. 6:**
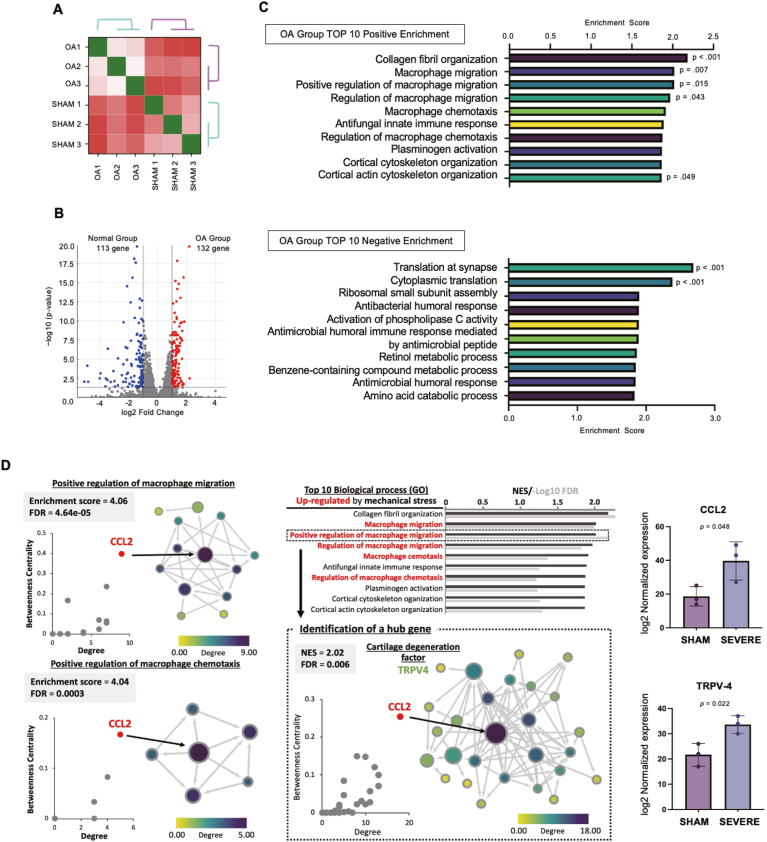
Transcriptomic and functional characterization of ankle osteoarthritis (OA) induced by graded instability. (A) Heatmap showing the overall correlation and clustering of DEGs among groups. (B) Volcano plot of DEGs of the osteoarthritic cartilage and normal ankle cartilage (criteria: false discovery rate <0.05; |log_2_FC| > 1). Red and blue dots represent upregulated and downregulated genes in osteoarthritic cartilage, respectively. (C) Gene Ontology Biological Process enrichment analysis performed using Gene Set Enrichment Analysis revealed distinct biological signatures in upregulated (top) and downregulated (bottom) gene sets. Upregulated genes were mainly enriched in pathways related to extracellular matrix organization and macrophage migration, whereas downregulated genes were associated with translation and metabolic processes. (D) A network analysis identified key hub genes within the OA-related network, including *CCL2* and *TRPV4*, which were closely connected to cartilage degeneration and mechano-inflammatory signaling. Node color indicates degree centrality, and node size reflects betweenness centrality. Corrected expression levels using count data significantly increased for CCL2 and TRPV4.

A transcriptomic analysis revealed distinct gene expression profiles of the ankle joints with osteoarthritis and sham-operated ankle joints (Fig. 6A). Hierarchical clustering clearly separated the two groups, indicating consistent transcriptomic alterations associated with mechanical instability. A volcano plot analysis identified 245 differentially expressed genes (false discovery rate <0.05; |log_2_FC| >1), including 132 upregulated and 113 downregulated genes in the osteoarthritis group (Fig. 6B). The top 10 upregulated and downregulated genes are summarized in Table 1, Table 2. A Gene Ontology enrichment analysis using Gene Set Enrichment Analysis showed that the upregulated genes were significantly enriched in pathways related to extracellular matrix remodeling and inflammatory activation, including collagen fibril organization, macrophage migration, and plasminogen activation (Fig. 6C). In contrast, the downregulated genes were mainly involved in protein synthesis and metabolic processes, such as cytoplasmic translation and ribosomal subunit assembly (Fig. 6C). Furthermore, a network analysis identified key regulatory nodes within the osteoarthritis-related gene interaction network (Fig. 6D). Among these, *CCL2* was identified as a central inflammatory mediator, whereas *TRPV4* was recognized as a mechanosensitive factor that links altered mechanical loading with inflammatory signaling and cartilage degeneration. Consistent with the findings of this exploratory analysis, relative expression levels of Trpv4 and Ccl2 were higher in the SAI group than in the sham group.

**Table 1:** Top 10 upregulated genes in osteoarthritic cartilage.

Symbol	log_2_FC	Adjusted p	Regulation (osteoarthritic vs. normal)
Fcrl2	2.212	0.003	Up (OA↑)
Gdf5	2.184	<0.001	Up (OA↑)
Loxl2	1.853	<0.001	Up (OA↑)
Crispld2	1.783	<0.001	Up (OA↑)
Tesc	1.712	<0.001	Up (OA↑)
Il22	1.710	0.027	Up (OA↑)
Tlr2	1.705	<0.001	Up (OA↑)
R3hdml	1.6965	<0.001	Up (OA↑)
Ldlr	1.695	<0.001	Up (OA↑)
Tbxas1	1.685	<0.001	Up (OA↑)

**Table 2:** Top 10 downregulated genes in osteoarthritic cartilage.

Symbol	log_2_FC	Adjusted p	Regulation (osteoarthritic vs. normal)
Prg2	−5.082	0.009	Down (OA↓)
Stfa2	−4.808	0.009	Down (OA↓)
Rdh8	−4.674	<0.001	Down (OA↓)
S100a8	−4.008	0.003	Down (OA↓)
Defa5	−3.952	<0.001	Down (OA↓)
Np4	−3.588	<0.001	Down (OA↓)
S100a9	−3.468	<0.001	Down (OA↓)
Retnlg	−3.442	<0.001	Down (OA↓)
Acta1	−3.247	0.009	Down (OA↓)
Camp	−2.986	0.007	Down (OA↓)

## 4 Discussion

In this study, we established a rat model of graded ankle instability by sequentially transecting the ATFL and CFL to induce varying degrees of ligament injury. Our results showed that greater mechanical instability of the ankle joint led to more pronounced cartilage degeneration and functional impairment. These findings suggested that joint instability plays an important role in the early cartilage changes, thus providing a useful experimental framework for investigating the mechanical basis of post-traumatic joint degeneration.

The relationship between joint instability and cartilage degeneration has been reported by several previous studies. Tochigi et al. [5] used a rabbit model and reported a clear correlation between anterior drawer displacement and the degree of cartilage degeneration. Similarly, Murata et al. [6] used a rat model and demonstrated that increasing anterior drawer displacement was associated with cartilage degradation. Moreover, stabilization of joint instability was associated with reduced cartilage degeneration, osteophyte formation, and the expression of catabolic enzymes such as MMP-13 [7,19,20]. Clinically, patients with osteoarthritis frequently report joint instability, and subjective instability has been correlated with the extent of cartilage damage [21,22]. Regarding the ankle joint, Liu et al. reported a model in which mechanical instability of the ankle–subtalar joint complex was induced by transection of the CFL as well as either the ATFL or the deltoid ligament [23]. As a result, the model exhibited impaired balance, shortened stride length, and increased cartilage wear, which suggested progression to PTOA. Similarly, Saliba et al. described a model involving surgical excision of the ATFL and CFL that produced mild cartilage damage without affecting the subchondral bone [24]. However, graded reproduction of mechanical instability and its quantitative correlation with cartilage degeneration have not yet been fully characterized.

This study demonstrated an association between ankle joint instability and cartilage degeneration. Furthermore, a correlation between ligamentous laxity and histological cartilage degeneration suggested that abnormal mechanical loading after ligament injury may also disrupt cartilage homeostasis in the ankle, as shown in knee models. However, the rate of degeneration in our model was relatively mild compared with that in knee PTOA models [25]. Additionally, the OARSI scores were lower than those observed in intra-articular knee models. This difference may be attributable to preservation of intra-articular structures in the present ankle model, which induced predominantly mechanical instability without intra-articular damage. Therefore, by reproducing mechanical instability without joint invasion, this model serves as a useful experimental system for analyzing the tissue-level consequences of altered joint mechanics. Consequently, the graded ankle instability model may be considered a relatively mild and physiologically relevant experimental model of osteoarthritis.

The changes observed in this study may represent an early-stage osteoarthritis phenotype rather than advanced or irreversible degeneration [26]. Early osteoarthritis is defined as a predestructive phase characterized by subtle surface irregularities, early extracellular matrix disorganization, and mild activation of inflammatory signaling changes that may remain reversible if mechanical balance is restored [27]. Such a stage is particularly relevant to the clinical progression of osteoarthritis after ligament injury. An RNA sequencing analysis further suggested molecular alterations that may be associated with mechanical instability. The upregulated genes in osteoarthritic cartilage include *Fcrl2*, *Gdf5*, *Loxl2*, *Crispld2*, *Tesc*, *Il22*, *Tlr2*, *Ldlr*, and *Tbxas1*, which are functionally linked to inflammatory signaling, extracellular matrix remodeling, and metabolic adaptation. In particular, *Gdf5* [[28], [29], [30]] and *Loxl2* [31] are involved in matrix remodeling and fibrosis, whereas *Tlr2* [32,33] participates in immune-mediated and cytokine-mediated inflammatory pathways that promote cartilage degeneration. Furthermore, *CCL2* was identified as a key inflammatory mediator that promotes macrophage infiltration, whereas *TRPV4* acts as a mechanosensitive ion channel that links abnormal mechanical stimuli to inflammatory signal transduction [[34], [35], [36]]. The coordinated activation of these factors suggests the presence of a CCL2–TRPV4 axis that may contribute to the transition from mechanical stress to inflammatory cartilage degeneration. This molecular network should be considered hypothesis-generating and represents a potential mechanobiological pathway involved in early osteoarthritis progression rather than a definitive mechanism. Accordingly, further functional validation is required to determine whether targeting this axis can effectively modulate disease progression.

This study had several limitations. First, the observation period was limited to 8 weeks postoperatively, and longer-term joint remodeling and subchondral bone changes require investigation. Therefore, this study focused on early-stage osteoarthritic changes. Second, the posterior talofibular ligament in the ankle OA model was not transected. Although the posterior talofibular ligament may play a stabilizing role in the rat ankle that differs from its role in humans [37], it was intentionally preserved to avoid excessive joint destabilization. Therefore, the biomechanical contribution of the posterior talofibular ligament was not analyzed. Third, the transcriptomic analysis included a relatively small sample size, which limited the statistical power and reproducibility of the RNA sequencing results; therefore, the transcriptomic findings should be interpreted as exploratory and hypothesis-generating. Finally, because of interspecies differences, sex differences, and age, the complex anatomy and loading characteristics of the human ankle cannot be fully replicated in the rat model.

## 5 Conclusions

We established a rat model of graded ankle instability by performing sequential transection of the ATFL and CFL without disrupting intra-articular structures. As a result, we observed that increased mechanical instability was associated with more severe cartilage degeneration and functional impairment, suggesting a relationship between ligamentous laxity and cartilage degeneration. Additionally, this model reflected relatively mild and gradual progression of osteoarthritic changes, which may be useful when investigating early-stage disease mechanisms related to mechanical instability. This experimental system may also provide a basis for future studies of the transition from CAI to ankle osteoarthritis as well as for exploring preventive or therapeutic approaches that target mechanically induced joint degeneration.

## Author contributions

KM and SK contributed equally to this work as co-first authors. KM was responsible for the overall experimental design, study conception, and execution of experiments. SK primarily conducted the experiments and data collection. NS, CT, and K. Nihei performed the histological analysis and evaluation of cartilage specimens. The sequence analysis was performed by KM. The bioinformatics analysis was conducted by SK. NK made a major intellectual contribution to the preparation and foundation of the manuscript together with KM. All authors discussed the results, contributed to the preparation of the manuscript, and approved the final version.

## Availability of data and materials

The data supporting the findings of this study are available from the corresponding author upon reasonable request.

## Declaration of generative AI in scientific writing

The authors used generative AI tools (e.g., ChatGPT) solely to improve the readability and language quality of the manuscript. All content was subsequently reviewed, verified, and edited by the authors to ensure accuracy and scientific integrity. In addition, the manuscript underwent professional English editing by a native speaker via Editage (https://www.editage.com/).

## Funding

This work was supported by 10.13039/501100001691JSPS KAKENHI (grant number 25K03070).

## Declaration of competing interest

All authors declare no competing interests.
